# Whisker Movements Reveal Spatial Attention: A Unified Computational Model of Active Sensing Control in the Rat

**DOI:** 10.1371/journal.pcbi.1003236

**Published:** 2013-09-26

**Authors:** Ben Mitchinson, Tony J. Prescott

**Affiliations:** 1 Department Of Psychology, The University Of Sheffield, Sheffield, United Kingdom; Queen's University, Canada

## Abstract

Spatial attention is most often investigated in the visual modality through measurement of eye movements, with primates, including humans, a widely-studied model. Its study in laboratory rodents, such as mice and rats, requires different techniques, owing to the lack of a visual fovea and the particular ethological relevance of orienting movements of the snout and the whiskers in these animals. In recent years, several reliable relationships have been observed between environmental and behavioural variables and movements of the whiskers, but the function of these responses, as well as how they integrate, remains unclear. Here, we propose a unifying abstract model of whisker movement control that has as its key variable the region of space that is the animal's current focus of attention, and demonstrate, using computer-simulated behavioral experiments, that the model is consistent with a broad range of experimental observations. A core hypothesis is that the rat explicitly decodes the location in space of whisker contacts and that this representation is used to regulate whisker drive signals. This proposition stands in contrast to earlier proposals that the modulation of whisker movement during exploration is mediated primarily by reflex loops. We go on to argue that the superior colliculus is a candidate neural substrate for the siting of a head-centred map guiding whisker movement, in analogy to current models of visual attention. The proposed model has the potential to offer a more complete understanding of whisker control as well as to highlight the potential of the rodent and its whiskers as a tool for the study of mammalian attention.

## Author Summary

The management of attention is central to animal behaviour and a central theme of study in both neuroscience and psychology. Attention is usually studied in the visual system (most often using cats or primates) owing to the ease of generating controlled visual stimuli and of measuring its expression through eye movement. In this study, we develop a model of the expression of attention in another sensory modality, that served by the tactile whiskers of small mammals (such as rats and mice). This sensory system has long been a popular model in neuroscience and is well characterised. It has become recognised in recent years that the modulations of whisker movements prevalent in the behaving animal represent “active sensing” (in the sense of moving the sensors to optimise sensing performance), yet a unified understanding of these modulations is still lacking. Our model proposes just such a unified understanding, suggesting that whisker movement modulations can be understood as an overt expression of the animal's changing focus of attention. This proposal, therefore, offers to provide both an enhanced understanding of the whisker sensory system and an insight into the management of attention in these animals.

## Introduction

A succinct summary of contemporary models of primate visual spatial attention is that exogenous signals (those arising from external stimuli) from multiple sensory modalities and endogenous signals (those arising from internal processes) compete and combine to produce a spatial map of salience from which a single region of immediate spatial attention is chosen [1]–[3]. In the case of overt attention, this location is ‘foveated’ by the rapid re-positioning of the eyes with movements of the head and body following as necessary [4]. If multiple salient locations are present, they are visited sequentially. The degree and nature of integration between overt and covert attention (that expressed only internally), exogenous and endogenous influences, and inputs from different modalities are all matters of debate, as is a definition of attention itself [3]–[8]. One aspect, however, is uncontroversial: that overt attention is expressed by rapid orienting movements that centre the foveal region of the eye on the attentional target. Many small mammals, including laboratory rats and mice, possess in addition to vision a complementary and well-characterised sensory system driven by tactile stimulation of prominent arrays of sensitive whiskers, particularly those located around the snout [9]. Here, we will consider whether the movements of these whiskers might also represent an expression of overt attention, revealing areas of proximal space that are of high salience to the animal. Potentially, such a model would be useful to experimentalists interested in mammalian attentional processes and their neural substrates, not least owing to the growing ease with which observations of whisker movement and position can now be made and analysed in these animals even when they are freely behaving.

Whisker movements have been most studied in animals that express ‘whisking’, a periodic protraction and retraction of the whiskers, typically occurring at several cycles per second (each cycle being termed a ‘whisk’) and in bouts lasting several seconds, with a close coupling of the oscillatory motions of the left and right whisker fields. Most data have been gathered using rats [10]–[12], though analyses are also available for mice, shrews, opossums and hamsters [13]–[16]. Many studies have now described significant departures from spectrally pure, bilaterally symmetric and synchronous whisking, revealing that both spatial and temporal parameters of whisker movements are under active control and can change rapidly in response to environmental conditions as well as to the motivations of the animal [12], [17]–[24]. Furthermore, small changes in whisker position can lead to large changes in sensory signals [25]–[27]. Thus, the proposition that an understanding of whisker movement is a pre-requisite to an understanding of whisker sensory signals has become a key focus of research [28]–[33]. This shift has been facilitated by the increasing availability of experimental tools for measurement of whisker movements [18]–[20], [34] as well as for automated analysis of large high-speed video datasets [34]–[37]. Not only is whisker movement of interest to the researcher who wishes to understand whisker sensory processing (and sensory systems in general), but these movements may also provide data about the internal state of the animal [32], [38]. Since whisker motion can be modulated when the head is stationary some useful measures are available also in the head-restrained condition [17], [39].

The modulation of whisker motion parameters under different conditions has been previously explained as arising from reflex responses (e.g. [19], [20], [40]) or from task-specific sensing strategies (e.g. [12], [41]). Furthermore, computational models developed by the current authors and evaluated in biomimetic whiskered robots [42]–[45] have demonstrated that a mix of positive and negative feedbacks, such as could plausibly be mediated by brainstem loops [46], can produce some of the observed whisker modulations. However, a simple reflex model cannot explain all modulations—for instance, those driven by conditioning [47], [48] or anticipation [17], [18], [20], [23], suggesting the involvement of higher centres in motion modulation [49]. Below, therefore, we motivate and develop a new model of whisker movement control that has as its key variable the region of spatial attention. The explicit representation of this region, as a tactile ‘salience map’, represents a significant departure from current theories and our own earlier models of whisker control, and provides a theoretical bridge to the current paradigm for understanding visual attention in primates, in which salience maps are a core concept [6]. We go on to reprise three behavioural experiments in simulation using the new model and report comparable results to those obtained using animals [19], [20], [23] using analyses closely replicating those employed in the original studies. In our discussion, we summarize the key features of the model and of our results, compare it with competing models and discuss its limitations, suggest experiments that might invalidate it, and discuss its likely neural substrate. In addition, we highlight two architectural features common to any model of this form. Thus, this report both represents a step forward in our understanding of active sensing in rodents and highlights the potential of the rodent and its whiskers as a tool for the study of mammalian attention.

## Methods

**Figure 1:**
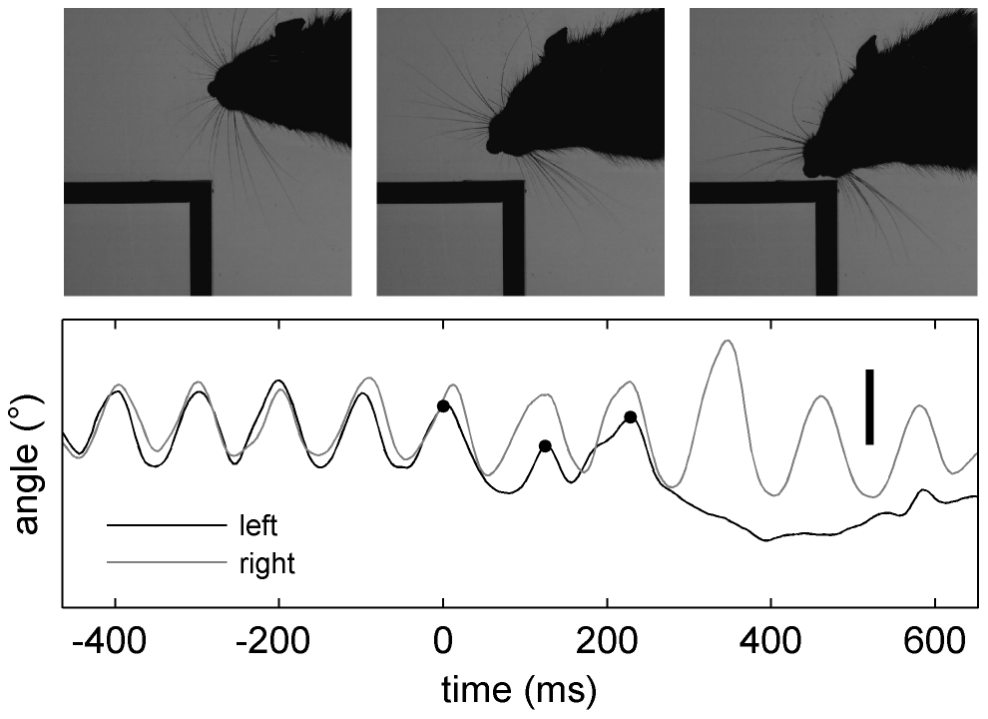
Rat behaviour. (Top) Three still frames from a top-down video recording of a rat encountering and orienting to the corner of a square object with vertical walls (data from [20]). Each successive frame is at approximately the time of maximum protraction of three consecutive ‘whisks’ (t = 0 ms, 120 ms, 230 ms)—the first is that immediately following the rat's first contact with the object. Two behavioural responses can be seen in the subsequent frames: (i) the whiskers are positioned asymmetrically around the snout and (ii) the tip of the snout is brought to the point of contact with the object. The whole video (covering the same time range as the plot) is available as Video S1. (Bottom) Average bilateral protraction angle of the whiskers recovered from the same video over a time period covering the encounter (left/right is black/grey; vertical scale bar has length 30°). Main feature of these signals until contact at t = 0 ms is periodic protraction and retraction known as ‘whisking’. The times of the three still frames are marked as dots on the trace from the left hand whiskers (see main text).

The upper panel of Figure 1 (and Video S1) shows the behaviour of a rat as it approaches, detects, and orients toward an object. This top-down view displays the most prominent degree of freedom of each whisker: rotation around the follicle (at the base of the shaft) resulting in ‘sweeping’ of the whisker rostro-caudally with the largest component of movement being in the horizontal plane [22], [50], [51]. Typical unperturbed periodic whisking can be seen in the first half of the trace of average bilateral whisker protraction angles shown in the lower panel of the figure. The current study focuses, however, on the modulations of periodic whisking that occur in response to environmental and internal conditions as illustrated, for instance, in the second half of the trace where whisking becomes strongly bilaterally *asymmetric* in response to contact with the object. Whisker positioning is, of course, dependent on head position, therefore our model also addresses the issue of moving the head and body in order to reposition the whiskers on larger spatial and temporal scales [52]. The model will not directly address variability in the periodic component of whisker motion, which can also be modulated (e.g. [21]), or the extension to three dimensions, although both of these topics are considered in the discussion. To explain the development of our model we next summarise some of the key observations of rat whisking behaviour that motivated its development together with some of the earlier functional explanations these observations gave rise to. We then operationalize the attentional hypothesis underlying the new model, and provide a detailed description first in conceptual form, then in terms of its implementation as a computer simulation, also explaining how the model will be evaluated in comparison to biological data.

### Motivation for the model

We have previously shown that whisker motion in the horizontal plane can be well summarized by just two variables for each side of the snout [23]: mean (across whiskers) angular position (henceforth, ‘mean protraction angle’) and the angular position difference between caudal and rostral whiskers (henceforth, ‘angular spread’). Several distinct observations of correlations between these and other behavioural variables have been reported. An early result in rat, that whisker protraction angles increase as the animal approaches the location of an anticipated contact [11], [17], [18], [20], has been recently matched and quantified in mouse [24]. Two further observations first made in rat have also been extended to mouse and opossum [15]. The first, which we term Head-Turning Asymmetry (HTA), is that mean protraction angles are adjusted to be more caudal/rostral on the side of the animal into/away from a future turn of the head [15], [19]. The second, Contact-Induced Asymmetry (CIA), is the observation that mean protraction angles are adjusted to be more caudal/rostral on the side of the animal near/away from a nearby object [15], [20] (see also Figure 1). A further observation is the Rapid Cessation of Protraction (RCP) that interrupts the protraction phase of a whisk movement when whiskers on one side of the animal make contact with an obstruction [20], [23]. We use the term Spread Reduction (SR) for the observation that the angular spread on each side of the snout is reduced during contact with objects in the vertical plane versus non-contacting whisks [23]. Finally, recent work in our lab has shown that animals engaged in rapid (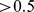 m/s) goal-directed locomotion employ tonic protraction (increased mean protraction angles and a reduced amplitude of periodic whisker movement, [53]).

To account for the observation of HTA, Towal and colleagues proposed that the whiskers search in the space into which the head will shortly be moved, perhaps partly to avoid collisions [19]. To account for contact-driven observations (RCP, CIA, SR) we proposed the general control strategy of ‘Minimal Impingement, Maximal Contact’ (MIMC, [20], [23], [42]) whereby whiskers are controlled so as to maximize the number of contacts but avoid excessive whisker bending within each contact (minimizing impingement). In addition, we recently hypothesized that tonic protraction during rapid forward locomotion reflects a strategy for collision avoidance whereby the ‘look-ahead’ distance of the animal is maximized [53]. Here, we propose that a single mechanism may be sufficient to explain all of these observations, including responses to anticipated contact.

### Development of the model

One clue to the nature of this mechanism is the observation that unilateral contact often elicits head-turning towards the contact point suggesting that CIA (Contact-Induced Asymmetry) and HTA (Head-Turning Assymetry), at least, may be related. The simplest possibility is that they are examples of the same response, to head movement or whisker-contact, expressed under different circumstances, but this is excluded by the following two cases. First, CIA is expressed regularly even where head-turning is precluded or absent, such as when the animal is following a wall ([20]; Video S1, S2, S3 all show examples of CIA in the absence of head-turning). Second, and conversely, HTA is expressed in the absence of any contact [19]. Nonetheless, these observations may be related through a hidden variable. In the case of HTA, whisker asymmetry precedes head-turning; therefore, unless whisker asymmetry drives head-turning directly—which seems unlikely—a hidden variable is implied.

**Figure 2:**
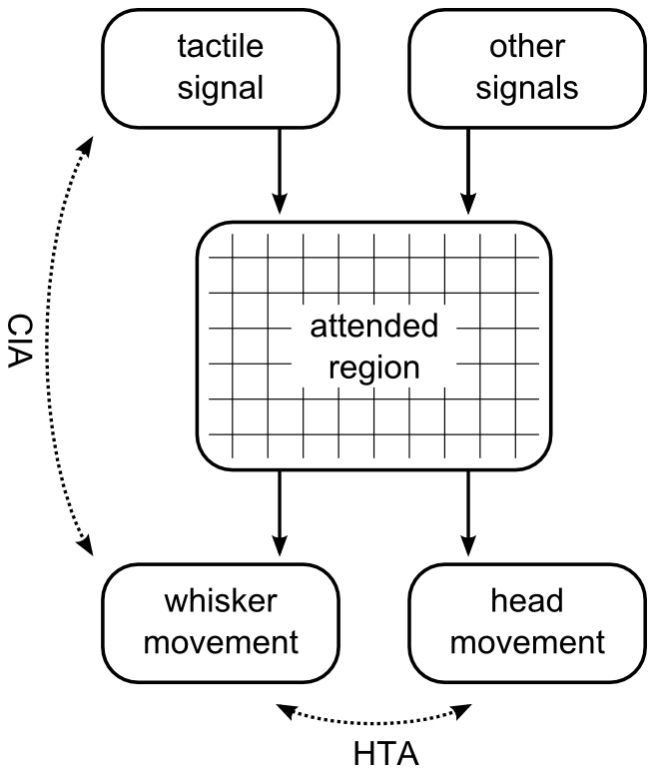
Model. Solid arrows indicate causal influences. Multiple influences affect the attended spatial region. One key influence will be whisker-environment contact (‘tactile signal’); others will include data from other sensory modalities and endogenous influences (‘other signals’). The ‘attended region’ drives both ‘whisker movement’ (rapidly and consistently) and ‘head movement’ (on a longer timescale, and only when this is not precluded by local geometry). Dotted lines show relationships that have been observed in animals. CIA is a correlation between contact and asymmetry in whisker movement. HTA is a correlation between turning of the head and asymmetry in whisker movement.

Seeking this hidden variable, we ask: Why does unilateral contact often elicit head-turning? The intuitive answer is that contact will often elicit attention, and attention will typically elicit orienting. We hypothesize, accordingly, that the hidden variable relating these observations is the ‘attended region’—that region of the external world which is currently the subject of the animal's attention—which can be affected by both tactile signals and other influences. According to this hypothesis, then, the mechanism underlying CIA is that laterally-biased contact generates laterally-biased attention which, in turn, drives asymmetric whisking, whilst that underlying HTA is that laterally-biased attention (however generated) drives asymmetric whisking and also head-turning. This model, summarised in Figure 2, is also consistent with observations of increased whisker protraction when contact ahead of the animal is anticipated and during goal-directed locomotion, both of which are conditions in which we might expect the attention of the animal to be focussed to the fore. Furthermore, the model explains why CIA is not observed in response to contacts in cases where the animal does not subsequently indicate attentiveness by orienting towards the contacted object [20].

**Figure 3:**
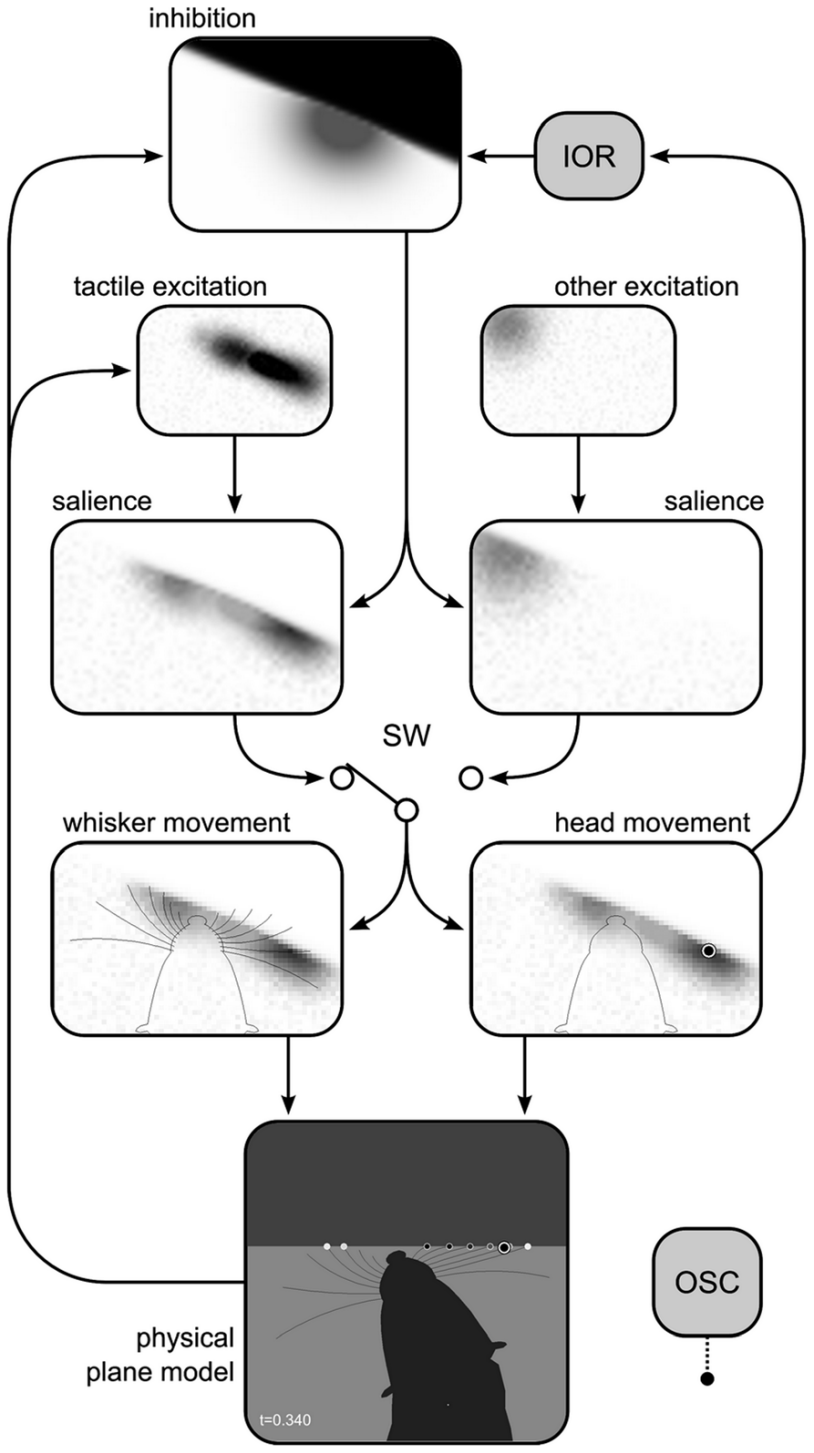
Implementation of the model. Boxes indicate components, solid arrows indicate causal influences. Extends/modifies model of Figure 2 with implementation-specific components: attended region made explicit as salience map(s); ‘other signals’ implemented as an endogenous stochastic source; inhibition, including a contribution from inhibition-of-return (IOR) system; oscillator (OSC); ‘physical plane model’ simulates mechanics. Separate salience channels are maintained for tactile and ‘other’ signals and selected at switch (SW). *Data snapshot*. Within the boxes is displayed a data snapshot from a point of maximum protraction during a whisk against a vertical surface. Physical plane model in world-centric coordinates includes head, whiskers and obstacle surfaces; whisker contacts are shown as dots (darker dots indicate stronger bending) and current target of foveation as a ‘target’ icon. Tactile and ‘other’ signals are mapped into head-centric excitation maps which drive salience maps (darker areas represent higher salience; pattern corresponding to wall can be seen in tactile salience map). Activity in salience map regions inside obstacles as well as in previously-visited regions (IOR, see text) is inhibited. Tactile salience channel is selected at SW owing to higher peak salience than ‘other’ channel. Whisker movement panel shows maximum protraction computed to roughly achieve MIMC with respect to attended region. Head movement panel shows current target of foveation (target icon) at peak of salience map. Video S4 shows the operation of the implementation during a trial including this snapshot (which was taken at t = 0.340).

Thus, our central hypothesis is that a transformation from the attended region to whisker protraction angles is the primary driver of long-term modulations of whisker movement (that is, on timescales longer than that of a single whisk cycle). The second behavioural response seen in Figure 1, the orienting of the snout tip, also intuitively appears to be an expression of overt attention since this movement serves to reposition a generalised sensory ‘fovea’—a body region in which are located the microvibrissae, lips, teeth, tongue, and nose, [9], [54], [55]—as well as an important actuator for small mammals: the jaws. We have, therefore, previously argued that movement of the head driven by switches in spatial attention represents a very significant component of the exploratory behaviour of small mammals ([42], [44], [56], [57]; see also [55]). Therefore, in analogy with the literature on the behaviour of visual animals, we refer to discrete head movements delineated by attention switches as ‘foveations’. The current model ties together these two modes of expression of attention, using a single representation of the attended region—in the form of a ‘salience map’—to drive movements of both the whiskers and the head (and, consequently, of the body). The remainder of this section details our implementation of this model, starting with an overview, and continuing with sub-sections detailing each computation, the headings of which correspond to the labels on the boxes in Figure 3.

### Model Implementation

#### Overview

The current model is focused on the movement of the whiskers and head, as driven by tactile and other (i.e. non-tactile) stimuli. In this section, we present an implementation (Figure 3) that simulates the model, the movement of the whiskers in a two-dimensional environment and their deflection against simulated obstacles, and the resulting tactile sensory signals, thus closing the sensorimotor loop and providing for the simulation of behavioural experiments. This implementation represents the attended region of space explicitly in the form of salience maps covering the area around the snout tip (120 by 80 mm discrete grid with element size 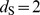 mm). Whilst this tactile sensorimotor loop offers a simple model of the driving of spatial attention by tactile signals, it does not emulate the ‘other signals’ of Figure 2 since simulation of non-whisker sensory systems and of intrinsic systems that drive orienting (such as motivation) are outside the scope of the model. Nevertheless, it is necessary that the simulation generate plausible sequences of gross behaviour if we are to make ethologically-relevant observations of it. To this end, the implementation includes a number of additional components, as follows. First, in order to motivate exploratory behaviour in the absence of tactile sensory input we include a stochastic mechanism to generate nearby attentional targets—the ‘other signals’. This mechanism is a proxy for the far richer motivational systems and other sensory modalities—olfaction, vision and audition—that would contribute to exploratory behaviour in the animal. Second, in order to avoid perseveration whereby the model repeatedly orients to the same position in space, we include a spatial memory system that implements ‘inhibition of return’ (IOR). Whilst it is recognised that biological IOR is ‘a complex, object-based and dynamically adaptive process’ [1], we follow a similar practice to some contemporary models of visual attention [58] and generate IOR through a relatively simple mechanism that is not intended to correspond directly to the underlying biological mechanisms. Finally, as a proxy for the interactions between multiple systems that lead to periodic gross behaviour in rats [9], [32], [59], we include an oscillator that ticks regularly (every 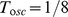 s) to drive periodic behaviour (specifically, whisking and switches in the spatial focus of attention).

The remainder of this sub-section details the operation of the components of the implementation, and its structure reflects that of Figure 3. We begin, in the next paragraph, by explaining why two salience maps are used to represent a single ‘attended region’. We go on to describe the loops that are illustrated in the figure, component by component. Since several parts of this description rely on an understanding of the morphology of the simulated animal, we begin by describing the component that specifies this morphology, the ‘physical plane model’. Thus, our description starts and ends with the signals that are passed to the physical plane model, the control variables for movements of the whiskers and the head. The parameters in the text are the ‘Reference’ values from Table 1; the effect on our results of varying these parameters to the ‘Adjusted’ values is reported in our sensitivity analysis, below. The discrete-time implementation, developed in Mathworks Matlab, is Euler-integrated with sample time 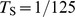 s.

**Table 1:** Parameters of the implementation.

Name	Description	Reference	Adjusted
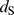	spatial resolution	2 mm	1 mm
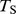	temporal resolution	1/125 s	1/250 s
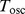	oscillator period	1/8 s	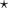
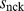	fovea-neck separation	50 mm	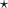
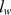	whisker length	44 to 8 mm	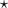
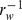	whisker curvature	−0.01 to 0.08 mm^−1^	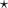
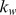	whisker sensing gain	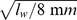	1.5 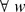
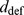	deformation meas. distance	5 mm	3, 10
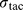	tactile excitation width	8 mm	4 mm, 12 mm
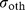	‘other’ excitation width	20 mm	10 mm, 30 mm
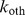	‘other’ excitation gain	0.5	0.25, 0.75
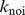	excitation noise gain	0.025	0.010, 0.100
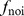	excitation noise bandwidth	8 Hz	4 Hz, 12 Hz
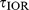	IOR memory length	4 s	1 s, 10 s
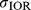	IOR width	20 mm	10 mm, 30 mm
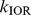	IOR gain	0.5	0.25, 0.75
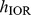	IOR max magnitude	0.66	0.50, 1.00
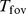	foveation period	0.175 s	0.125 s, 0.250 s
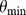	min protraction angle	30°	10°, 50°
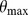	max protraction angle	175°	150°, 180°
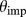	impingement angle	0°	−10°, −20°
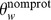	nominal protraction angle	75° to 145°	45–160°, 100–130°
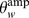	protraction amplitude	30° to 45°	15–30°, 45–60°
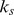	activity gain	2	1, 3
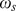	activity exponent	2	1, 3
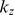	caudal bias base	500	250, 1000
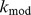	modulation strength	0.50	0, 0.25, 0.75, 1.00
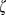	protraction duty cycle	70%	50%, 80%
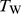	whisking dynamic period	0.025 s	0.010 s, 0.050 s

‘Reference’ values are those used in the experiments reported in Results. Parameters with subscript 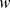 are whisker-specific, and specify a range of values 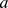 to 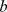; these parameters vary linearly in the specified range between caudal (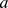) and rostral (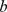) whiskers. ‘Adjusted’ values are used in our sensitivity analysis; adjustments of some parameters (marked 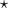) were not considered (see main text for details).

#### Two salience maps

Some perceptual tasks face an agent, whether biological or simulated, with solving what Treisman (1996) [60] called the ‘part binding’ problem. That is, binding together the parts of an object, as distinct from the background and parts of other objects, into a single unit. The management of spatial attention may be intimately linked with the solution of different types of binding problem [61]; in any case, segregation of stimuli into distinct objects is a pre-requisite, by definition, of selecting one object as the target of attention. In our implementation, we might collate information from exogenous and endogenous sources (‘tactile excitation’ and ‘other excitation’, Figure 3) in a single spatial representation of salience; we could then use a plausible neural mechanism (such as ‘winner-take-all’) to mediate competition for attention, selecting a single spatial location to focus on (e.g. [62]). However, we would have to solve the part binding problem if we were to recover distinct candidate target regions (that is, targets with spatial extent). This non-trivial problem is not the subject of this study, so we avoid this complexity by maintaining two independent salience maps, one for each class of signal (tactile and other), additionally ensuring that each map contains only one possible target of attention at any one time (see below). Selection of the target of attention, then, amounts to selection of one of the maps, and it is the activity in the selected map that drives whisker and head movement. The data snapshot in Figure 3 shows the choice between a tactile salience signal corresponding to a sensed wall and an example of the stochastic signal. The map with the higher peak value—in this case, the tactile signal—is selected at the switch (SW).

#### Physical plane model

**Figure 4:**
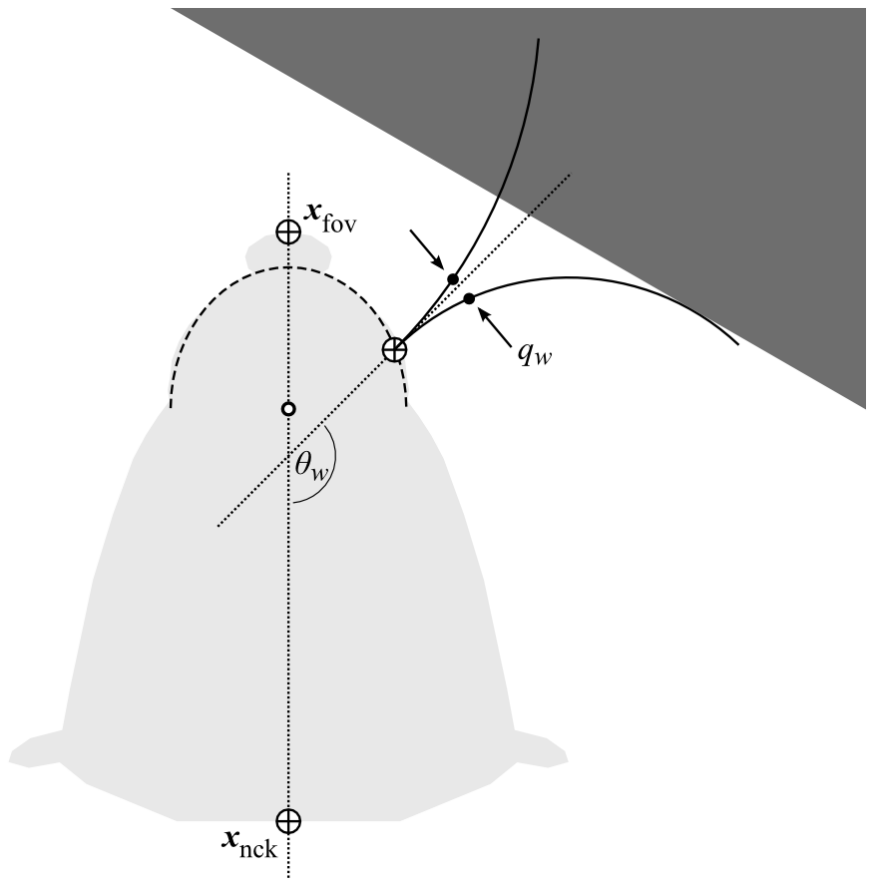
Plane model detail. Head (light grey) location/orientation is defined by 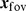 and 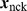. Nearby obstacle (dark grey). Whisker base is located on ‘mystacial pad arc’ (dashed curve) which traces the snout outline (open dot marks arc center). Whisker shaft angle at the base, denoted 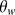, is defined with respect to head midline (dotted lines). Unperturbed whisker arc (upper solid line) intersects obstacle. Perturbed whisker arc (lower solid line) is found by adjusting curvature caudally until no intersection occurs. Deviation of point marked with solid dot from unperturbed to perturbed arc is denoted 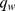.

The physical plane model (Figure 4) simulates the movements of head and whiskers in an environment that can be populated with rectangular obstacles. The head of the animal is represented by the locations of the neck joint, 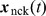, and of the tip of the snout (which we refer to as the ‘fovea’), 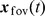, with 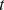 the sample time. Initial conditions are 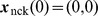 and 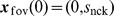, for a fovea-neck separation of 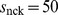 mm. 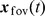 is an input to the physical model (specified below); 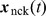 is moved at each sample along a straight line towards 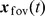 to maintain the fovea-neck separation. The ‘mystacial pad arcs’ define the base locations of the whiskers—these are ellipsoidal and lie along the snout outline (see Figure 4). The locations of the bases of the whiskers (seven on each side) are laid out along these arcs with linear spacing. Whisker length (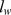; 44 to 8 mm, caudal to rostral) and unperturbed whisker curvature (somewhat rearward to somewhat foreward, caudal to rostral, see Figure 3) are based on anatomical data [9], [63]. The protraction angle of the 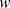
th whisker, denoted 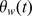, is the angle made between the base of its shaft and the midline of the head. The unperturbed arc of the 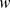
th whisker at time 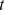, then, is defined by its length and curvature, its base location (derived from 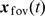 and 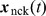), and its protraction angle 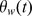. Thus, the controllable degrees of freedom of the plane model are 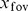 and the 14 whisker base angles, 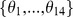. Whisker bending against obstacles is then simulated quasistatically: the curvature of each whisker is adjusted to be increasingly caudal until it just does not intersect any of the rectangular obstacles in the plane model (if no obstacles are nearby, the curvature under bending, thus, is left at the unperturbed curvature). An afferent *contact signal* for each whisker is then computed through a procedure mimicking that used in physiological investigations of whisker afferent responses to whisker bending (e.g. [26]), as follows. First, the deviation under bending, 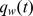, from the unperturbed position of a point some distance 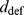 (5 mm, following [26]) along the arc from the whisker base is measured. The contact signal for the 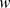
th whisker is then computed according to 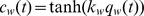, with 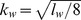 a whisker-specific gain, and the function 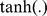 providing a saturation effect. A value of 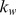 proportional to square root whisker length was chosen heuristically to approximately normalise the strength of the contact signals experienced by each whisker during the experiments, which otherwise tended to be weaker on the longer whiskers. Thus, the magnitude of the (positive) contact signal 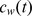 reflects the degree to which the 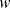
th whisker is deformed at time 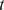; 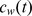 can be read from the shading of the dots indicating whisker contact in figures and videos, from white (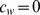) through to black (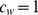)—see Figure 3, for example. If 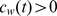, the location of the intersection between the whisker arc and the obstacle placing the tightest constraint on curvature (i.e. the obstacle causing whisker bending) is recorded as the *contact location*, 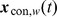. A component representing the animal body, seen in some videos, is included solely to aid visualisation and does not affect computation.

#### Tactile excitation

The contact signals 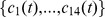 are mapped into head-centric space at the contact locations 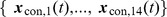 at each sample period. Each contact contributes additively to activity in the corresponding location of a head-centric tactile input map 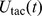 through a Gaussian spatial filter of width 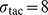 mm and height 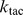 (the filter width defines something akin to the spatial ‘resolution’ of the system). Over-unity entries of 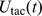 are then set to unity. Thus, 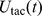 is populated by ‘blobs’ of activity in the contact locations when contact is occurring, and is empty otherwise. In order to use historical data to drive behaviour, sensory data must be stored; the transient nature of afferent information in the whisker sensory system owing to the periodic motion of the whiskers only serves to underline this need. Thus, we implement a tactile excitation map 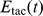 with memory, implemented as a leaky-max operation. To maintain the spatial validity of historical data we use a dynamic remapping scheme to compensate head movement that is functionally the same as that proposed by Dominey & Arbib (1992) [64]. We implement the dynamic remapping using an image processing function, 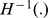, which compensates the movement of the head. Thus, the iterative update for activity in the tactile excitation map 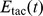 (Figure 3) is written
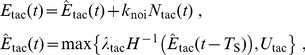
(1)where 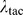 is a decay term, 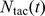 is an array noise source where each entry is a coloured Gaussian random process with unity variance in the 0-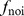
Hz band, and 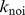 is the noise gain. The operation 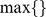 is applied entry-wise to sets of matrices, so that 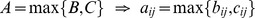. In practice, the application at each sample period of the lossy image processing operation 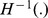, which includes re-sampling of the transformed information onto the original discrete grid, has a side-effect of fairly rapid decay in the state of 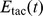, so that the parameter 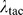 is superfluous and we can set it to unity.

#### Other excitation

The computation for the ‘other’ (non-tactile) channel is similar. At each oscillator tick, a single random location contributes to activity in the other input map 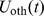 through a Gaussian spatial filter of width 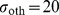 mm and height 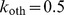, so that a single blob is formed; when the oscillator does not tick, 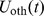 is empty. Then, the update for activity in the other excitation map 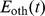 (Figure 3) mirrors Equation 1:
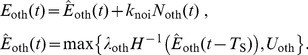
(2)

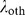
 is set to unity for the reason given above for parameter 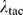, and 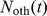 is an equivalent process to 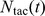. The arrangements of simulated obstacles used in the experiments, below, do not include narrow physical channels (since the experimental set-ups we are modelling also lacked narrow channels), so that only one contiguous object can be present in the tactile excitation map at any one time. Each pattern generated in the ‘other’ salience map explicitly consists of only one region of activity. These arrangements ensure that each excitation map contains only one possible target of attention at one time, as outlined above.

#### Inhibition and salience

An inhibition map, 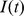 (Figure 3), is created from two components. The first component, 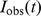, implements absolute inhibition inside any obstacles, avoiding physically impossible foveations, and side-stepping the shortcomings of the very simple physical plane model. 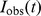 has unity activity inside and behind (from the fovea's point of view) obstacles and zero activity elsewhere. The second component, 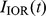, implements inhibition-of-return through partial inhibition of previously-visited locations using a loop closely akin to that presented by Itti *et al.* (1998) [62]. As each head movement completes (i.e. at the subsequent oscillator tick), the current fovea location is added to an allo-centric set of visited locations, 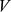, and any location not visited for more than 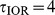 s is removed from 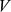. Each location in 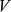 is mapped into head-centric space and contributes additively through a Gaussian filter of width 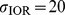 mm and gain 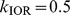 to 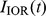. The inhibition map is then given by
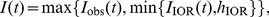
(3)where the operation 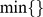 is applied entry-wise to sets of matrices and 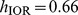 is a parameter limiting the maximum inhibition from IOR. The two salience maps, denoted 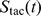 and 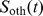, are then computed in the same way, according to
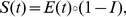
(4)where 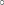 denotes the entry-wise (Hadamard) product operation. The map 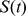 with the higher maximum value is re-selected at each oscillator tick (that is, the position of switch SW in Figure 3 is set), and is denoted 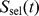. 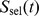, thus, encodes the ‘attended region’ at time 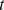, and drives the head and whisker movements.

#### Head movement

At each oscillator tick, a new target for foveation 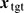 is chosen at the location of the peak in 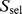, to be reached after the foveation period, 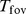. An open-loop minimum-jerk trajectory is then pre-computed between the current fovea location, 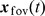, and its future location, 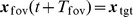 (with zero velocity at each end). Note that if 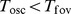, therefore, each head movement (foveation) is interrupted before it completes, and the fovea only reaches all the way to foveation targets that are selected on consecutive ticks.

#### Whisker movement

The central computation of the model is a transform that generates a maximum protraction angle for each whisker, 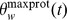, based on the activity in the selected salience map, 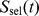. Since we cannot, in general, infer the attended region of an animal, the data that would be required to recover this transform automatically from biological data are lacking. In their absence, we assume a transformation based on MIMC [20], which dictates that as many contacts as possible should occur, but that they should be ‘light’. An animal could learn such a transform through trial and error during the post-natal period [65]; here, we construct it by hand. In words, the maximum protraction angle for each whisker is chosen, as far as possible, such that the whisker ‘just enters’ the attended region. An example of the result of this transform is shown in Figure 3, panel ‘whisker movement’. Instantaneous protraction angles, 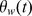, then vary periodically between this controlled maximum protraction angle, 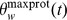, and the minimum protraction angle (which is set to the maximum angle minus a fixed amplitude parameter). This periodic variation—whisking—is driven by a signal derived from the oscillator so that the point of maximum protraction occurs at the oscillator tick (whisking, therefore, is at 8 Hz which is a typical frequency in rats, [15]). The transform is now defined mathematically.

The *e*th entry of salience map 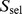, denoted 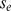, represents a region centred on a location 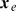. For locations where it is possible to do so, a protraction angle for this location and the 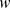
th whisker, 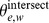, is computed such that the unperturbed whisker (i.e. the whisker with its parametrized curvature) would intersect 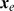. The ‘proposed’ maximum protraction angle for this location/whisker is then computed according to
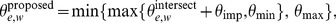
(5)where 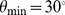 and 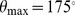 are the smallest and largest allowed protraction angles, respectively, and 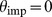 is a fixed ‘impingement’ parameter. For locations that the whisker cannot intersect owing to its length (those further away from the whisker base than the whisker tip), 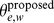 is set to 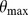 so that whiskers tend to ‘reach’ forward if they cannot contact any part of the detected object. At each sample time, then, a weighted sum is computed to arbitrate between the angles proposed by each active entry of 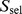. 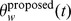 is given by
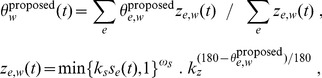
(6)where 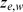 gives more weight to active entries (first term) and to entries that propose more caudal protraction angles (second term). Thus, 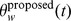 tends to position the whisker so that it reaches the *first* part of the active region in the map that it would reach during a protraction (the parameters 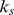 and 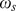 govern how activity is interpreted, whilst 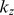 controls the degree to which more caudal angles are weighted).

Two factors affect how strongly the instantaneous maximum protraction angles, 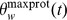, are affected by those proposed, 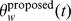. The first is the contrast in 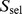, defined as 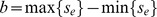, with 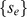 the set of all entries in 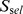. The second is the modulation strength parameter, 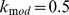. For each whisker, we compute
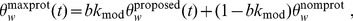
(7)where 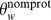 (linearly spaced from 75° caudal to 145° rostral) is the nominal protraction angle for the whisker.

#### Whisking

**Figure 5:**
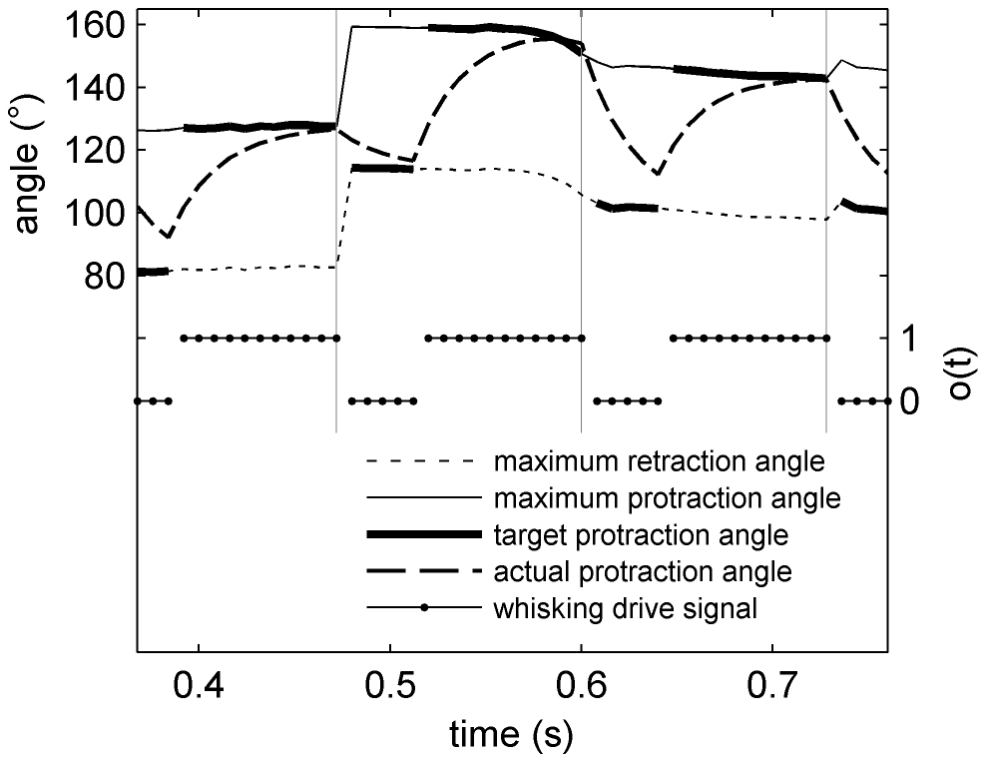
Whisking pattern generation. (Lower trace, axis to right) Solid line marked at each sample with dots is whisking drive signal, 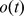. (Upper traces, axis to left) Thin dotted and solid lines indicate maximum retraction and protraction angles (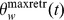 and 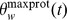), respectively, for one whisker (the most rostral whisker on the left). Overlaid thick lines show the target protraction angle, 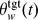, which is equal to 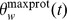 or 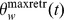 depending on the value of 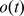 (see Equation 9). Feint vertical lines show the time of oscillator ticks (times of falling edges in 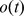). Actual whisker protraction angle, 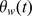, is indicated by the dashed line and is driven towards 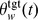. A sharp increase in maximum protraction angle occurs shortly before 0.5 s; this change is reflected in the whisker protraction angle most strongly during the subsequent protraction which ends at around 0.6 s.

Finally, we construct the instantaneous whisker protraction angle for each whisker, 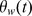, based on the maximum protraction angle derived above, 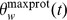, the whisker-specific whisking amplitude parameters, 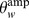, and timing information from the oscillator. We define the whisking drive signal 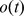 which is zero in the first (100-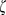)% of each oscillator cycle, and unity in the remaining 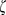
%, so that its falling edge coincides with the oscillator tick (see Figure 5). The (instantaneous) maximum retraction angle is defined as
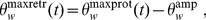
(8)where 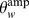 varies linearly from 30° (caudal) to 45° (rostral). Then, 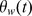 is driven towards 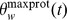 when 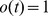, and towards 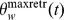 at other samples, according to
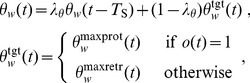
(9)where 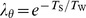 defines the shape of the periodic whisker movement trajectory.

### Behavioural methods and their simulation

Below, we use computer simulation of our attentional model to reprise three earlier behavioural experiments. In each case, we position obstacles in the simulated environment, allow the model to control the whiskers and head for some period, and make the following measurements. First, we measure the location of the tip of the snout over time, 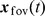, and the head bearing (that is, the angle of the head midline that runs from the neck joint 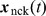 to 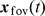). Second, we record the *measured* protraction angle of the 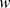
th whisker, 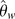, according to the methodology we have used previously in the behavioural laboratory [23]. That is, we locate the base of the whisker, and a point two thirds of the way along its shaft, and derive the angle between the vector connecting these points and the head midline. Similar strategies were used in most of the other behavioural work with which we make comparison [15], [19]. We go on to obtain the instantaneous mean protraction angle of all the whiskers on each side of the snout, 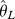 and 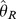, by simple arithmetic mean across the whiskers, again following precedent from analyses of behavioural data [15], [19], [23]. As a measure of whisker protraction angle that is unaffected by bending of the whiskers against obstacles, we also record the protraction angle at the base of the 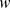
th whisker, 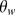, and compute the corresponding bilateral mean protraction angles, 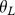 and 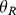. Presented examples of animal behaviour (stills and videos) were drawn from our archive of behavioural data to illustrate the text; recording methodology was described previously [20], [23]. Bilateral mean protraction angle presented in Figure 1 was recovered from the video data using the BIOTACT Whisker Tracking Tool (bwtt.sourceforge.net) and the ViSA tracking algorithm suite [37].

## Results

Above, we described an implementation of a new model of snout and whisker motor control as well as additional simulated components to permit observations of the model. In summary, this implementation (Figure 3) shares the basic form of models from the visual system literature (see [1] for a review)—that is, it includes a spatial map, bottom-up drive from the sensory periphery, non-specific top-down drive, inhibition-of-return (IOR), and outputs that drive overt attention. Experimental control over the model is exercised by choosing the location of any obstacles and the initial position of the head in a given trial. We have included only very simple models of motivation and IOR sufficient to generate patterns of exploratory behaviour, both around and away from obstacles, that can be compared to those seen in animals. In particular, in the absence of obstacles, foveation is driven only by a random signal, and the head model expresses stochastic exploratory-like behaviour (for instance, see Video S5). When obstacles are present, foveation is also driven by contact (for instance, see Video S6). The interaction between foveation to the points of contact with obstacles and inhibition of recently-visited locations leads to thigmotaxis—specifically, the fovea tends to follow obstacle contours and a form of ‘wall-following’ behaviour emerges. Maximum whisker protraction angles are controlled according to a transform driven by the current region of spatial attention and inspired by the ‘Minimal Impingement, Maximal Contact’ (MIMC) hypothesis [20]. In this section, we use this system to reprise three earlier behavioural experiments showing evidence for active touch sensing strategies in the rat—head-turning asymmetry (HTA), contact-induced asymmetry (CIA) and spread reduction (SR). For each study, data are extracted from the simulated model to emulate as closely as possible the original analyses of high-speed digital video recordings of behaving animals.

### Simulated behavioural experiments

#### Head-turning asymmetry

**Figure 6:**
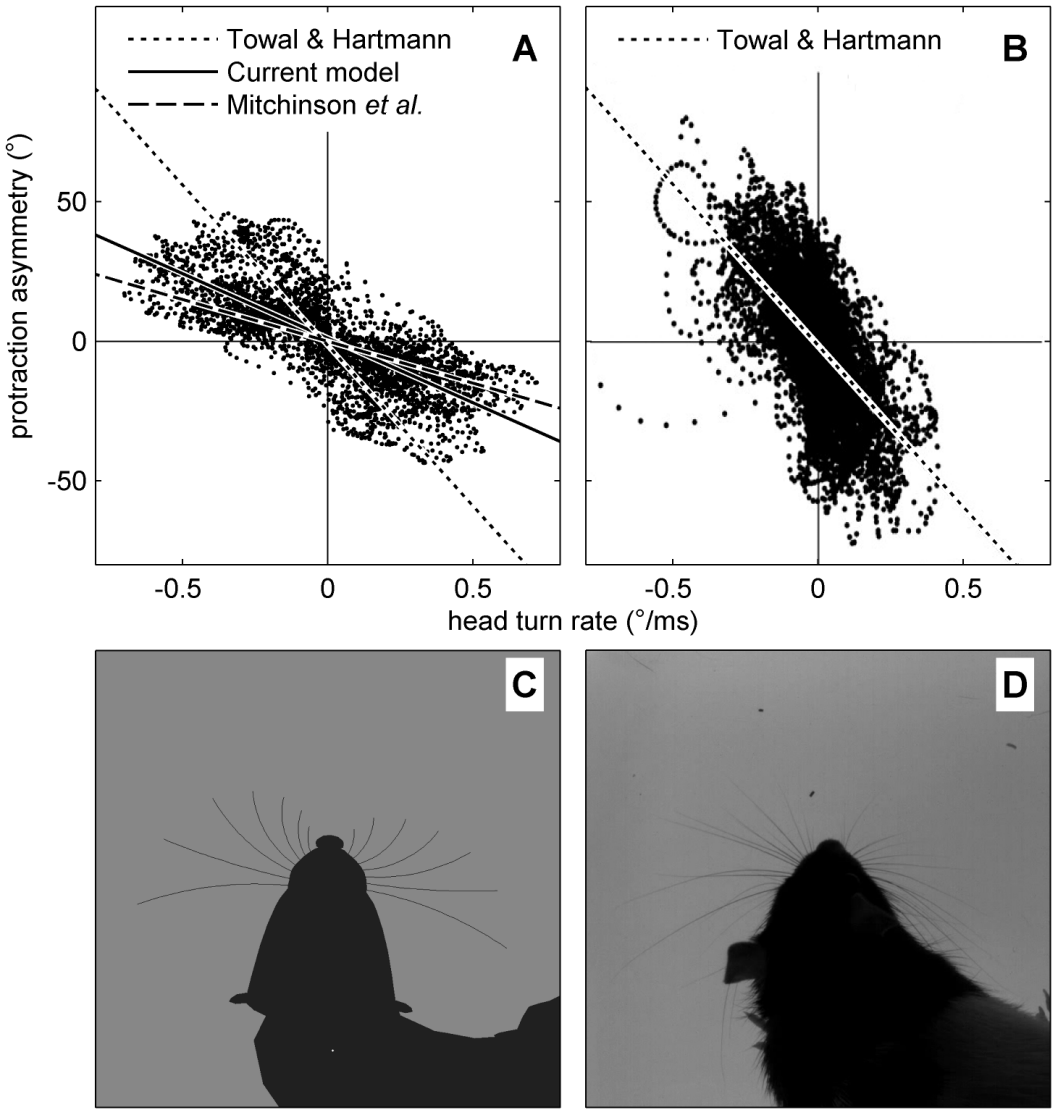
Head-turning asymmetry. (A) Results from model. Each dot represents one sample; solid line is line of best fit. Also shown are lines of best fit from analogous observations made by Towal & Hartmann (2006) [19] (dotted line, their Figure 6a) and Mitchinson *et al.* (2011) [15] (dashed line, their Figure 4a(i)). Note, therefore, that results from simulated model fall between results from two behavioural studies. (B) Results from Towal's & Hartmann's behavioural experiment [19], reproduced with permission. (C/D) Stills from model (C) and behavioural experiment (D) showing asymmetry in bilateral protraction angles during head turn to the right. Still in (C) is taken from Video S5.

Measurement of HTA has been previously reported in rat during motivated whisking in free-space by Towal & Hartmann (2006) [19] and during non-motivated whisking above a floor by Mitchinson *et al.* (2011) [15]. To reprise those experiments, we place no obstacles in the environment such that spatial attention is driven only by the stochastic input; thus, the fovea makes a sequence of orients to random locations. We run the simulation for thirty seconds, and measure the ‘left minus right’ asymmetry between the bilateral mean protraction angles, 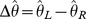. This is plotted, in Figure 6A, against the instantaneous head-turning rate. The figure can be fairly directly compared with Figure 6a from Towal & Hartmann (reproduced in our Figure 6B)—Towal & Hartmann measured motivated whisking in free space (i.e. with no floor present)—and with Figure 4a(i) from Mitchinson *et al.* Lines of best fit from both of these studies are also included in our Figure 6A. Head turn rate correlates well with whisker angle asymmetry—that is, HTA is strongly expressed. The coefficient of the linear relationship is −46 (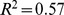); this compares with coefficients of around −115 (Towal & Hartmann) and −30 (Mitchinson *et al.*) in the behavioural analyses. Note that no obstacles are used in this experiment, and the physical model does not contain inertial terms, so no whisker deformation occurs. Therefore, the presented results are unchanged if computed using whisker base angles 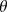 rather than perturbed whisker shaft angles 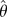.

#### Contact-induced asymmetry

**Figure 7:**
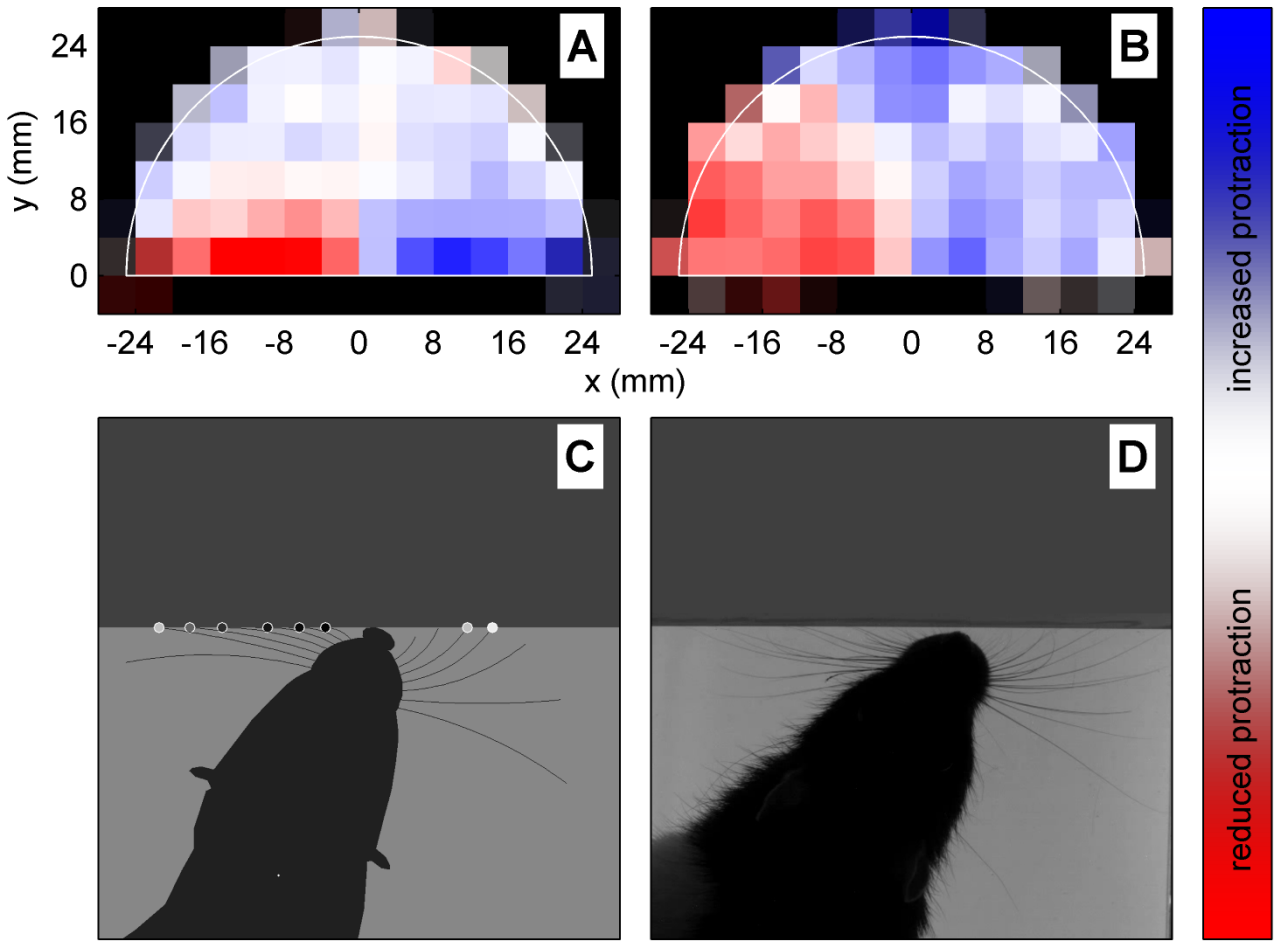
Contact-induced asymmetry. (A) Results from model (see text for analysis method). Mean protraction angle of the whiskers on the left (or right—see text) in NEAR relative to mean value in FAR, plotted against the binned location, 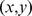, relative to the fovea of a single nearby wall (4 mm square bins). Red/white/blue indicates mean protraction angle is reduced/equal/increased relative to 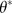, with full saturation for each colour indicating 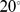 difference. White semi-circle indicates 25 mm from fovea at 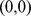, i.e. the region graphed in Figure 4c of Mitchinson *et al.* (2007) [20]. (B) Results from behavioural experiment (in rat, [20], their Figure 4c), re-analysed on a rectangular grid to match current analysis. Electromyogram strength in NEAR, rather than mean protraction angle, is graphed, relative to mean electromyogram strength in FAR; fully saturated red/blue indicates 33% difference. (C/D) Stills from model (C) and behavioural experiment (D) showing asymmetry in bilateral protraction angles driven by encounter with angled surface. Still in (C) is taken from Video S6.

CIA has been reported previously in rats interacting with vertical walls by Mitchinson *et al.* (2007) [20] and in rats, mice and opossums interacting with vertical corners by Mitchinson *et al.* (2011) [15]. Here, we reprise the first experiment and its analysis, by constructing an ‘arena’ (400 mm square) and allowing the model to explore inside for one hour (simulated time). The time series 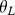 and 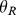 are recovered, along with the position of the neck and the nose, for each sample. These time series are low-pass filtered (2 Hz, zero-phase) before being down-sampled to 8 Hz, yielding approximately one sample per whisk. We then identify all samples (whisks) in which the nose was within 25 mm of one wall and at least 100 mm distant from all others (i.e. samples where exactly one wall was near enough to the snout to be contacted by the whiskers, 6799 whisks, ‘NEAR’ set), as well as those samples in which the nose was at least 100 mm distant from all walls (i.e. samples for which no whisker-wall contact was possible, 7230 whisks, ‘FAR’ set). FAR is used to obtain an ‘unperturbed’ average mean protraction angle: we computed the average value of 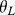 and 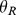 across all samples in FAR and both sides, to give this value, denoted 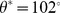. Next, within NEAR, and for each sample, we find the point on the nearby wall nearest the nose, and summarise the wall location by this point 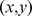 relative to the nose. Also for each sample, we obtain the *relative* mean protraction angle on the left, 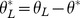. This provides a measure of the relative amount of protraction on the left hand side (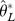) for an obstacle in the position 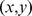. This relative protraction angle can then be graphed against obstacle position, as in Figure 7A. Finally, assuming the behaviour of the animal is symmetric, we mirror the similar data from the right side whiskers, and add it to the data for the left side whiskers—it is this pooled data that is used to generate Figure 7A.

Figure 7A can be most directly compared with Figure 4c from Mitchinson *et al.* (2007) [20]—Mitchinson *et al.* measured electromyogram rather than whisker movement and binned results on a radial grid. The result from that study is re-analysed on a rectangular grid to match the current analysis, and presented in our Figure 7B. Figure 7A can also be fairly directly compared with Figure 5b(i) from Mitchinson *et al.* (2011) [15]—Mitchinson *et al.* observed interactions with corners, rather than flat walls, and over short timescales.

The features of the plot of the behavioural analysis (Figure 7B) can be summarised as follows. First, the red/blue pattern on the left/right sides indicates that protraction angles are reduced/increased ipsilateral/contralateral to an obstacle—this is CIA. Second, the blue region in the middle at the top indicates that protraction angles are increased when an obstacle is present ahead of the snout—this corresponds to ‘reaching’ forward towards an obstacle, the presence of which is either sensed (using e.g. vision) or is anticipated (using memory). In comparison, the results from the model (Figure 7A) indicate robust expression of CIA (with similar magnitude to that reported in behavioural experiments), but no clear expression of forward reaching. This discrepancy is to be expected, since we have not included vision or memory in this implementation, so that objects located ahead of the snout rarely affect whisker movements in the model. Observation of the simulation underway—for instance, see Video S6 (around t = 18.84 or t = 19.64) or Video S4 (around t = 0.84)—confirms that, on occasions when the attention is switched away from a wall to the middle of the arena (i.e. from the contact input to the ‘other’ input) there is a brief period where the snout is near the wall but CIA is not expressed toward it, corresponding to our informal observation that CIA is not expressed towards apparently non-attended objects [20]. In this experiment, the use of perturbed whisker shaft angles 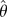 rather than base angles 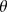 results in the measurement of physical deflections of the whiskers by the environment rather than a measurement of whisker control by the simulated system, so it is not informative.

#### Spread reduction

**Figure 8:**
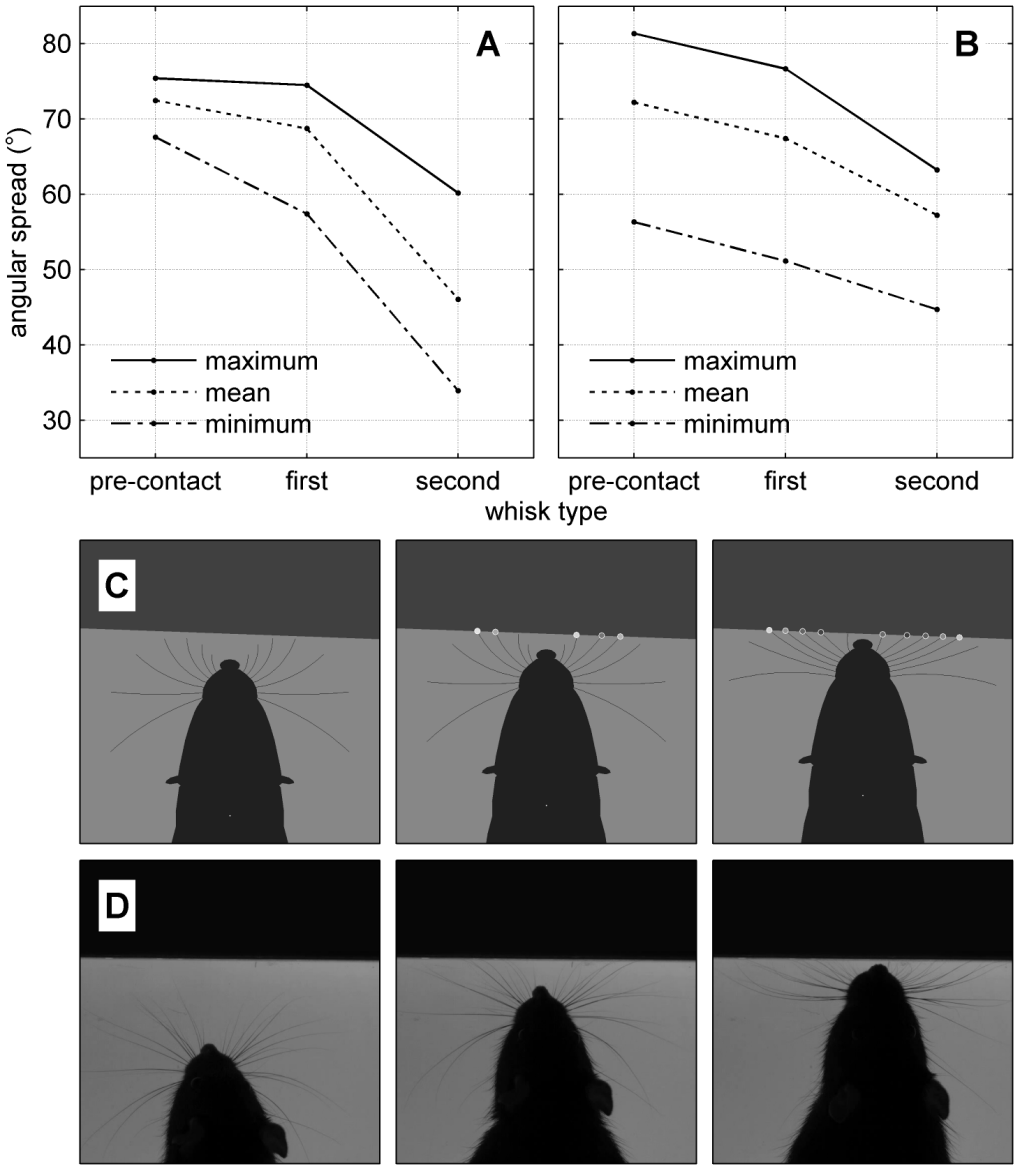
Spread-reduction. (A) Results from model. Solid/dotted/chained lines are maximum/mean/minimum spread within the whisk, against whisk type (see text). (B) Results from behavioural experiment (in rat, [23], their Figure 2b), data re-plotted. (C/D) Stills from model (C) and behavioural experiment (D) showing the trial condition of rat approaching vertical obstacle (three panels in each case show time of maximum protraction in pre-contact, first and second contact whisks). Stills in (C) are taken from Video S7.

We reprise the experiment of Grant *et al.* (2009) [23] by using a single obstacle representing a vertical wall and constraining the movement of the fovea so that the snout moves towards the wall in a straight line. The wall is angled at random between plus or minus ten degrees from perpendicular to the midline of the animal, and the constant approach speed is randomly chosen between 10 and 50 mm/s. One hundred trials were computed, for 200 potential samples of data from one or other side of the snout; 69 of these met the data selection criteria that Grant *et al.* defined. Following their analysis, we start by identifying the pre-contact, first and second contact whisks, in only these selected trials. In each time sample of each trial, we measure the angular spread as the angular separation between one caudal whisker (the rearmost) and one rostral whisker (the fifth from the rear), using 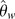. Then, in each identified whisk, we measure the minimum, maximum and mean spread across time. These values are averaged within whisk types and across trials, and the results are plotted in Figure 8A which can be fairly directly compared with Figure 2b from Grant *et al.* (reproduced in our Figure 8B)—Grant *et al.* used freely exploring animals, whilst in our simulated trials the model is constrained to approach the wall at a constant speed. As in the behavioural experiments, SR is expressed moderately in the first contact whisk and more strongly in the second. Again following Grant *et al.*, we performed a check to ensure that our results were not due primarily to the changing shape of the whiskers through deformation against the wall rather than to changing whisker control. We repeated the analysis using the whisker base angles (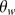) rather than the whisker shaft angles (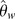): spread was lower overall, but the pattern of spread reduction was unchanged (results not shown).

### Sensitivity analysis

The results above can be summarised as follows. During exploration in free space, the simulation expresses HTA with a coefficient of linearity between those reported in two behavioural studies. During exploration near walls, the model expresses CIA with a strength comparable to that reported in two behavioural studies. During approach to a wall, the model expresses SR (some reduction in first contacting whisk, substantially more in second) with comparable strength to that reported in a behavioural study. To assess the sensitivity of these results to the ‘Reference’ parameter choices listed in Table 1, we realised the three experiments multiple additional times, making adjustments to one or a few parameters in each case, and assessing the results for the qualitative findings given above. We did not test adjustments to the parameters marked 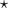 in Table 1 since these are fairly well-defined by previous reports (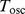 is a temporal scale parameter which defines only the overall rate of behaviour; the other three are anatomical parameters). The effect of adjustment of the remaining parameters is reported below.

To begin with, we tried flipping the array 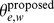 along the left/right dimension after it had been built. The asymmetries of HTA and CIA had their senses reversed, as expected, whilst the SR result was somewhat weakened, also as expected. Next, we checked that integration error was not affecting our results by using higher spatial (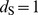 mm) and temporal (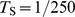 s) resolution; the CIA result appeared a little strengthened, but otherwise there was no effect. Similarly, most other adjustments to the parameters (listed in Table 1, column ‘Adjusted’) had only minor effects and did not change the qualitative results; those that did impact the results are now listed. Increasing all three width parameters (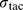, 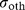, 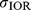) had little impact; decreasing them somewhat weakened the CIA result (though the main lateral bias remained robust). Raising 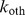 had little effect, but reducing it eliminated plausible gross behaviour in the CIA experiment so that the result could not be measured. Decreasing/increasing the excitation noise gain (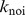) strengthened/weakened the results, as expected (at the high noise level, the SR result was qualitatively degraded). Decreasing 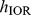 had little effect; increasing it had little effect on HTA or SR, and only slightly weakened the CIA result, apparently owing to changes in gross behaviour rather than any effect on whisker movement *per se*. Adjusting the nominal protraction angles 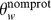 up or down affected the scaling just of the SR result, but did not change it qualitatively. Increasing the protraction duty cycle, 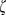, to 80% had little effect; reducing it to 50% introduced some noise into the CIA result (though the main lateral bias remained robust). Adjusting the overall modulation strength, 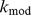, had the strongest effect of any of the tested adjustments, unsurprisingly—however, whilst the strength of all three results was very directly affected, all the results were qualitatively unchanged for all non-zero tested values. As expected, with a modulation strength of zero, both HTA and CIA plots are flat, whilst the SR plot shows a small reduction in spread owing to the measurement of physical whisker deformation.

## Discussion

The central variable of the model is a representation of the immediate region of space attended by the animal which rapidly modulates, through a fixed transform, the maximum protraction angles of the whiskers and drives the movement of the snout (specifically, the positioning of a generalised sensory ‘fovea’ around the mouth) on a longer timescale. Thus, both whisker and head movements are modelled as overt expressions of attention. In the implementation presented, the attended region is represented in the activity of a salience map driven by contact and by an endogenous stochastic signal and inhibited by an IOR mechanism, maximum whisker protraction angles are set by an MIMC-like transform driven by activity in the map, and the fovea is driven towards the location of the peak in the map. This implementation expresses HTA, CIA and SR, when challenged by experimental paradigms equivalent to those used in the behavioural laboratory. Furthermore, these results were robust to parameter variation—this is unsurprising, given the intuitive development of the underlying model presented in Methods.

### Whisking modulation as an example of rat cognition

Attention is a prototypical example of what is generally considered to be a cognitive process. That is, compared to the simpler notion of a reflex arc, attention requires mechanisms that can implement bottom-up filtering of stimuli, working memory for recent events, competitive selection, and top-down modulation (e.g. by motivational systems) (see, e.g. [66] for a review of the nature of attentional processing). Components that implement each of these computations are required to create even a relatively simple model of spatial attention as demonstrated by the model system we describe above. Whilst it is reasonable to seek simpler mechanistic explanations of a phenomenon such as the sensory modulation of whisker movement, there is evidence in a wide-range of domains—time [67]–[69], number [67], [70], reward [71], [72], decision-making [73], [74], space [75]–[78], and working and long-term memory [79], [80]—that rodents process information in a manner that reflects the operation of cognitive mechanisms sometimes approaching, in terms of their sophistication, those identified in primates. We propose that in the case of spatial attention, rat cognition again shares interesting similarities to primate cognition that have been largely overlooked (though, see [81], [82]). Specifically, that models of visual attention using salience maps, that have proved effective in explaining primate eye movement data, could have a useful analogue in the attentional mechanisms underlying rat vibrissal touch.

Whilst not a minimal model in terms of the computations involved, we propose that our attentional hypothesis for rodent whisking modulation is parsimonious in the sense of being explanatorily powerful. That is, the model accounts for multiple observed phenomena (HTA, CIA, SR), and, moreover, does so in a way that is robust to parameter change (see Sensitivity Analysis, above). The model should also naturally reproduce phenomena described in the literature that cannot, even in principle, be explained by reflex mechanisms. Specifically, anticipatory ‘reaching’, in the form of increased whisker protraction, has now been reported in a range of experimental paradigms: Sachdev *et al.* (2003) [17] reported unilateral reaching in anticipation of contact with a sensor that triggered a reward; Berg & Kleinfeld (2003) [18] reported reaching (alongside changes in temporal parameters) when animals were challenged to contact a discriminandum on the other side of a gap; our own observations of a freely-exploring condition also suggest reaching [20] (see Figure 7B) as does evidence of rats increasing whisker protraction during running [53]; finally, SR also appears to be anticipatory at least in part [23]. All of these experiments used rats, but reaching has also recently been observed in mouse by Voigts *et al.* (2013) [24], who highlighted that “The precision in amplitude modulation is not due to current sensory input” but rather relies on historical sensory information (i.e. on working memory).

The validity of the attentional explanation of whisking modulation can be further tested in the behavioural laboratory. One key prediction is that non-attended objects will not elicit whisker modulation, as we have previously observed informally in a handful of trials but have not yet quantified [20]. A possible preparation to test this prediction might be, for instance, a motivated animal seeking particular objects preferentially over others positioned nearby. A second key prediction is that whisker movement is modulated by spatial attention, however generated. A preparation for testing this might be an examination of the whisker movements of a head-fixed animal, with spatial attention manipulated by olfactory, auditory, or visual cues rather than by tactile stimuli. If, for instance, a whiff of an attractive odor from a specific direction elicited whisker movement toward that direction this would be strong evidence in favour of an attentional model of whisking modulation, in this case showing cross-modal transfer of target salience.

Our model does not include modulation of whisk frequency, nor changes in whisker movement at very short time-scales. As a result, two notable observations not accounted for by the model are Rapid Cessation of Protraction (RCP) [20], [23] and the ‘touch-induced pump’ (TIP) [40] both of which occur within the time-scale of a single whisk. As previously discussed [20], [83], these observations may reflect the operation of a rapid negative feedback loop, though alternative plausible models for RCP and TIP include (i) that they represent contact-driven changes in the timing of an underlying motor pattern and (ii) that they follow from rapid switches in spatial attention through the attentional mechanism proposed here (given the rapidity of responses in midbrain to whisker contact, [84]). Further experiments will be required to establish whether brainstem mechanisms alone are sufficient to elicit these phenomena.

### Neural substrates of tactile attention mechanisms

The model presented is abstract in form and also in substrate, however, neuroscientific evidence does point towards some likely substrates for different aspects of these attentional computations in the rat brain.

Most clearly, the superior colliculus (SC) would be a very plausible location for a spatial attention map to be sited. SC has the right inputs from somatosensory centres—rapid bottom-up inputs arrive directly from trigeminal sensory complex, whilst top-down inputs from somatosensory cortex are also present [84]–[86]—and the sensory organization is topographic [87]–[89]. More broadly, rodent SC receives inputs also from visual and auditory centres [90], reflecting that SC is an important centre for the integration of multi-sensory—specifically, spatial—information [91]. It also has the right outputs: it contains topographic motor maps for both orienting-like head movements [92] and apparently modulatory (non-periodic) whisker movements [93], [94] and has direct efferents to facial nucleus, the motor nucleus associated with the whisker musculature [85], [95]. Salience maps have been identified in SC [1] and it has been strongly implicated in the mediation of visual attention processing [96]–[99]. The proposal that SC plays a key role in rat orienting to whisker stimuli is consistent with its importance for rat prey capture [100]. Interestingly, adult-like HTA, CIA and SR emerge in the post-natal animal during overlapping periods in P12–16 [65], corresponding approximately to the time when SC is reported to be maturing anatomically (around the beginning of the third post-natal week, [89], [101]).

Aside from colliculus, other centres likely to be involved in attention management and/or whisker movement include motor cortex and the basal ganglia. Stimulation of vibrissal motor cortex (vMCx) can evoke whisking-like movements of the whiskers, and the parameters of stimulation affect the parameters of whisking [102]–[104]. In addition, motor cortex ablation significantly disrupts whisking parameters, particularly contralaterally [105]. These data suggest that vMCx is involved in initiating and modulating whisking even though whisking itself appears to rely on a CPG [32], [106], [107]. Activity recorded in vMCx during natural whisking reflects whisking onset as well as variations in amplitude and set-point, consistent with this hypothesis [108]–[110]. Interestingly, motor area M2 in rat has been analogised to the primate Frontal Eye Fields (FEF) [111], a key structure involved in primate oculomotor control and critical in relaying signals from frontal cortex related to voluntary control of visual attention [112]. In addition to projecting to the SC, the FEF, in primates, also project directly to the brainstem saccadic generator so that a primate with a SC lesion is still able to generate saccades. The M2 area in rat likewise has strong reciprocal connections with prefrontal cortex [113], projections to SC [114], and direct brainstem projections to areas involved in orienting [115]. Unilateral lesions in this area have been found to produce contralateral neglect in both primates and rats [111]. The basal ganglia (BG), in both rats and primates, are well-situated to gate switches of attention. SC, whisker somatosensory cortex S1, and whisker motor cortex, all project to similar regions of the dorsolateral striatum (DLS), the input region of the BG [116]. In the case of SC, the projection is via the thalamic intralaminar nuclei [117]. DLS then has an inhibitory projection to BG output structures including the substantia nigra pars reticulata which, in turn, tonically inhibits SC and, via the thalamus, areas of sensory and motor cortex related to the vibrissae, thus completing a double-disinhibitory loop that seems configured to select target representations that are of high salience to the animal [118], [119]. In primates, the role of BG in gating saccadic eye-movements to salient targets has been described in detail by Hikosaka *et al.* (2000) [120], and it seems plausible that the BG will play a similar role for whisker-guided orienting movement in rats.

### Architectural features of the model

The model has two interesting architectural features distinct to this system. First, whisker-centric data are mapped into a head-centric representation of space, implying dynamic routing of sensory data, in analogy to remappings of auditory and somatosensory data in other animals [91]. However, owing to the rhythmic exploration of space by the whiskers (along with inertial or contact-driven bending), the central representation of the periphery is constantly and rapidly on the move in such a model. In SC, rats have an approximately retino-centric topography in the superficial layers, whilst vibrissal data is represented in the deeper layers in spatial register with the overlying visual maps [87], [93]. At the same time, regions sensitive to stimulation of individual whiskers are large and overlapping under anaesthesia [86], [87], particularly in the rostral-caudal dimension, consistent with the large area of the visual field swept by individual whiskers as they move back and forth [50]. Whisker-sensitive cells in primary somatosensory cortex have been reported both to respond most strongly at particular whisker movement phases [121] and to encode whisker bending direction [122], and primary afferent cells that encode whisker phase have also been identified [27]. Thus, this highly dynamic model is consistent with existing data, whilst cells such as those identified could constitute part of a substrate for remapping, as has been previously discussed [27], [121], [122].

Second, whilst visual overt attention is primarily expressed through the azimuth and elevation angles of the eye [96], our model of tactile overt attention hinges upon the radial dimension since the generalised sensory fovea must be brought *to* an object rather than just pointed *at* it [9]. Accordingly, the current study could not have been performed without a representation of the radial dimension. In the current study, we did not represent the vertical dimension (primarily because behavioural data are lacking) but we routinely find it necessary to use three-dimensional representations of space as the substrate for spatial orienting in our work with robots (reviewed in [44]). The current proposal can be extended to three dimensions if a three-dimensional representation of the attended region is assumed, but whether extension in this way would respect the biological organisation remains an open and important question.

### Conclusion

In summary, then, our findings support the general hypothesis that there exists in the rat a system somewhat homologous to the visual orienting system known from primate studies [96], with the primary outputs being re-location of a generalised sensory fovea around the mouth, supported by body movements as required [92], and adjustment of the protraction angles of the whiskers, perhaps to favour a ‘Minimal Impingement, Maximal Contact’-like control aim. Within this system, superior colliculus may well play a key role, along with areas of cortex and the basal ganglia [111], [123]. This system probably forms only part of a larger system that generates whisker movements but most or all non-periodic components of motion may be mediated therein. Thus, this sensorimotor model has the potential to substantially improve our understanding of the modulations of periodic whisker movements that are observed in behaving animals. As highlighted recently by Schwarz *et al.* (2010) [124], a particular disadvantage of the head-fixed rat preparation is that the behavioural repertoire of rodents includes many whole-body movements, whisker movements being an exception. In contrast to widely-studied rodent attentional measurement paradigms (such as the 5-choice serial reaction time task, [125]), whisker movements could reveal attention on relatively short timescales, in considerable spatial detail, optionally in head-fixed preparations, with measurement remaining highly automatable. Thus, if whisker movements can be confirmed to reveal the region of spatial attention, their observation might provide a novel and practical tool for its investigation in small mammals.

## Supporting Information

Video S1Behaving animal (recorded at 250 fps, playback at 25 fps, slow 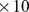). A top-down video recording of a rat encountering and orienting to the corner of a square object with vertical walls (see also Figure 1). Two behavioural responses can be seen: (i) the whiskers are subsequently positioned asymmetrically around the snout and (ii) the tip of the snout is brought to the point of contact with the object.(AVI)

Video S2Behaving animal (recorded at 250 fps, playback at 25 fps, slow 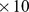). A top-down video recording of a rat encountering and orienting to the corner of a square object with vertical walls. After orienting, the snout becomes motionless, whisking slows and almost ceases, but bilateral asymmetry is maintained between the protraction angles of the whiskers on the two sides.(AVI)

Video S3Behaving animal (recorded at 500 fps, playback at 25 fps, slow 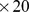). A top-down video recording of a sessile rat that is near to a rectangular object with vertical walls. Both snout and whiskers are nearly motionless, but strong bilateral asymmetry in whisker protraction angles is present throughout (1 second of recorded behaviour).(AVI)

Video S4Implementation (generated at 500 fps, playback at 25 fps, slow 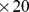). 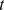 = 0.0 to 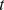 = 1.0 from an illustrative example of running the implementation. For details of the panels shown, see Figure 3 and its caption.(AVI)

Video S5(Simulated) Head-Turning Asymmetry (generated at 125 fps, playback at 25 fps, slow 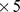). 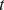 = 1.0 to 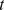 = 4.0 from the experiment HTA. Shows a top-down view of the simulated rat which is behaving freely in an experimental condition with no objects present. The rat is whisking and orienting to signals in the noise channel. The peak of the noise channel at each attention switch (and, therefore, the target to which the nose is brought) is indicated by the target icon. Bilateral asymmetry in whisking correlates with turning of the head.(AVI)

Video S6(Simulated) Contact-Induced Asymmetry (generated at 125 fps, playback at 25 fps, slow 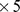). 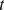 = 18.0 to 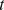 = 20.0 from the experiment CIA. Shows a top-down view of the simulated rat which is behaving freely in an experimental condition where a closed rectangular arena is present (the rat is inside this). The rat is whisking and orienting mostly to contacts with the arena wall. Bilateral asymmetry is driven by these contacts.(AVI)

Video S7(Simulated) Spread Reduction (generated at 500 fps, playback at 25 fps, slow 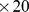). One trial from experiment SR. Data from both sides of the snout passed the inclusion criteria, in this case (no contact in pre-contact whisk, and at least two whiskers make contact in first contact whisk). Reduction in the spread between whiskers can be seen in the first whisk in which contact is made, but is much more marked in the second contacting whisk.(AVI)
